# Synthesis of Biomimetic Melanin-Like Multifunctional Nanoparticles for pH Responsive Magnetic Resonance Imaging and Photothermal Therapy

**DOI:** 10.3390/nano11082107

**Published:** 2021-08-19

**Authors:** Jing Qu, Devin Guillory, Pohlee Cheah, Bin Tian, Jie Zheng, Yongjian Liu, Courtney Cates, Amol V. Janorkar, Yongfeng Zhao

**Affiliations:** 1 Department of Chemistry, Physics and Atmospheric Science, Jackson State University, Jackson, MS 39217, USA; jing.qu@students.jsums.edu (J.Q.); dmcguillory@yahoo.com (D.G.); pohlee.cheah@jsums.edu (P.C.); tianbinphd@163.com (B.T.); 2 Mallinckrodt Institute of Radiology, Washington University School of Medicine, St. Louis, MO 63110, USA; zhengj@wustl.edu (J.Z.); yongjianliu@wustl.edu (Y.L.); 3 Department of Biomedical Materials Science, School of Dentistry, University of Mississippi Medical Center, Jackson, MS 39216, USA; ccates@umc.edu (C.C.); ajanorkar@umc.edu (A.V.J.)

**Keywords:** polydopamine nanoparticles, photothermal effect, pH-responsive materials, MR imaging

## Abstract

The design and development of multifunctional nanoparticles have attracted great interest in biomedical research. This study aims to prepare pH-responsive melanin-like nanoparticles for *T_1_*-weighted magnetic resonance imaging (MRI) and photothermal therapy. The new multifunctional nanoparticles (amino-Fe-PDANPs) are synthesized by copolymerization of dopamine and its derivative amino-N-[2-(diethylamino) ethyl]-3,4-dihydroxy-benzenepropanamide (N-Dopa) at room temperature. The size of nanoparticles can be controlled by NaOH concentration. The incorporation of N-Dopa is characterized by NMR and FT-IR. From transmission electron microscopy (TEM), the nanoparticles exhibit excellent dispersion stability in water and are spherical in shape. The MRI measurement has demonstrated that amino-Fe-PDANPs have a significant signal enhancement in responding to the acidic solution. Confirmed by the photothermal study, the nanoparticles exhibit a high photothermal conversion efficiency. The melanin-like multifunctional nanoparticles integrate both diagnosis and therapeutic functionalities, indicating the potential for theranostic application.

## 1. Introduction

Polydopamine (PDA) is a synthetic analog of natural melanin which is widely distributed in many parts of the human body, including hair, skin, and the iris of eyes [1,2]. Melanin-like nanoparticles are the ideal multifunctional platform for biomedical applications. They have been used in various applications such as tissue engineering [3,4], biosensors [5], and diagnosis [6,7]. Due to the aromatic rings on their surface, they can serve as drug delivery carriers, loading chemical drugs on their surface through π–π interaction and hydrogen bonding [8]. One of the most important properties of melanin-like nanoparticles is that they are completely biocompatible. It has been shown that melanin-like nanoparticles can be degraded into non-toxic components in living systems [9].

In addition, melanin-like nanoparticles have been used as efficient photothermal agents [10]. Photothermal therapy (PPT) is a non-invasive treatment strategy, which relies on a transducer to absorb and convert light energy into heat. The elevated temperature will stimulate hyperthermic responses and cause a hazardous effect on cells. Currently, non-degradable metallic nanoparticles such as Au [11,12], Ag [13], and Pd [14] are the commonly studied transducers for PTT as their structures can be tuned to absorb light in near IR range and enable deep tissue penetration. Metallic nanoparticles cannot be metabolized in the body, therefore melanin-like nanoparticles are a promising alternative when the translation study is considered.

More interestingly, melanin-like nanoparticles can also be used for magnetic resonance imaging (MRI) due to the ability to chelate with metal ions, making it possible to combine treatment with diagnosis [6]. The advantage of magnetic resonance imaging (MRI) lies in high-resolution function and anatomical imaging without being limited by the tissue penetration depth [15,16]. As radiation is not involved, MRI can be used to monitor the real-time changes of disease tissues during treatment. To date, most studies were focused on *T*_2_-weighted MR contrast agents [17,18]. In fact, *T*_2_-weighted magnetic nanoparticles usually suffer from a low signal-to-noise ratio. This is due to existing endogenous negative contrast in the body, for example, calcium depositions, bleeding, or the presence of other metals. In comparison, *T*_1_-weighted MR contrast agents can increase their longitudinal relaxivity to lighten the signal specifically. With regards to *T*_1_-weighted MRI contrast agents, extrinsic chelators such as DTPA or DOTA are usually needed to chelate Gd^3+^ or Mn^2+^ ions [19,20,21]. Because of the relatively low sensitivity of MRI, the high concentration of translation metals is still required for imaging.

To further improve the sensitivity of MRI, it is a promising strategy to develop environmentally responsive *T*_1_-weighted MRI contrast agents. The nanomaterials with stimuli response to physiological parameters such as reducing environment [22], temperature [23], pH [24], or enzyme levels [25] are found to be effective approaches to increase diagnostic sensitivity. Among them, pH stands out as an essential physiological parameter [26]. Specifically, dysregulated pH is identified as one of the hallmarks for tumors, where a “reverse” pH gradient across the cell membrane is spotted in tumor cells compared to normal cells. There are several different types of acid-responsive MRI agents based on polydopamine nanoparticles. Ge et al. synthesized Cu^2+^-loaded polydopamine nanoparticles for magnetic resonance imaging, reaching ~30% Cu^2+^ release rate in the slightly acidic environment [27]. Cheng et al. prepared the MnCO_3_@PDA that could serve well as an excellent MRI/PTT theranostic agent in an acid medium [28]. However, these pH-sensitive contrast agents are based on translation metals such as Mn^2+^, Gd^3+^, and Cu^2+^. There is a concern for the long-term toxicity [29].

Recently, the nanoparticles with tertiary amines were reported to exhibit nonlinear enhancement of fluorescent imaging in response to narrow pH change [30,31]. The nanoparticles based on tertiary amines have been demonstrated for sensitive disease imaging in responding to the acidic microenvironment [32]. We reason that polydopamine nanoparticles incorporating dopamine derivative with tertiary amine could lead to a sensitive pH-responsive MRI contrast agent. However, it is challenging to prepare nanoparticles from dopamine derivatives because of the reactivity and complexity of dopamine polymerization [33]. So far, there have been few reports on polydopamine nanoparticles incorporated with new dopamine derivatives.

In this paper, we plan to synthesize a new type of biodegradable multifunctional polydopamine nanoparticles copolymerized with dopamine derivative, amino-N-[2-(diethylamino) ethyl]-3,4-dihydroxy-benzenepropanamide (N-Dopa). We chose iron (Fe^3+^) as the magnetic metal ion due to its *T_1_* characteristics and minimal toxicity since it is a biological relevant element [34]. The nanoparticles will have good biocompatibility and respond to acidic environments due to the tertiary amine groups. The nanoparticles will exhibit high sensitivity for *T*_1_-weighted MRI and photothermal therapy properties after chelating iron (Fe^3+^).

## 2. Materials and Methods

### 2.1. Chemicals and Materials

Amino-N-[2-(diethylamino) ethyl]-3,4-dihydroxy-benzenepropanamide (N-Dopa, 95%) was purchased from Aldlab Chemicals (Woburn, MA, USA). Dopamine hydrochloride (C_8_H_11_NO_2_.HCl) was purchased from Sigma-Aldrich (St. Louis, MO, USA). Iron (III) chloride (98%) was purchased from Acros Organics (Fair Lawn, NJ, USA). Milli-Q water was used in this study.

### 2.2. Synthesis of Polydopamine Nanoparticles (PDANPs)

PDANPs were synthesized following a previously published procedure [35]. In a typical synthesis, Dopamine.HCl (22.2 mg) was dissolved in milli-Q water (10 mL). Then, NaOH solution (1 M, 115 µL) was added dropwise. The mixture was stirred for 4 h at room temperature. The product was purified by centrifuge (8000 rpm, 10 min) and washed with milli-Q water for three times. Finally, the solution was stored at room temperature for future use.

### 2.3. Synthesis of Amino-Fe-PDANPs

The as-prepared PDANPs (10.0 mg) and N-Dopa (0.7 mg) were added into a 10 mL round bottom flask and stirred vigorously for 1 h. Then the product was purified by centrifuge (8000 rpm, 10 min) for one time. After that, the solution is put back into the round bottom flask. Iron (III)-chloride (1.0 mg) was added into the flask and continue to react under stirring. After 3 h, the final product was separated by centrifuge (8000 rpm, 10 min) and then washed with milli-Q water for two times.

### 2.4. Characterization

^1^H NMR spectra were obtained using a Varian 500-MHz spectrometer with dimethyl sulfoxide (DMSO)-d6 as solvent and tetramethylsilane (TMS) as the internal standard. The Fourier transform infrared (FTIR) spectra were recorded on a Nicolet 10 (Perkin Elmer, Waltham, MA, USA). FTIR spectra were collected in solid state and taken from 400 to 4000 cm^−1^ with a resolution of 4 cm^−1^ for 64 scans. The surface morphology and size characterizations were performed using transmission electron microscopy (TEM, JEOL JEM-1011 (JEOL Inc, Peabody, MA, USA)) and scanning electron microscope (SEM, TESCAN LYRA3) (TESCAN, Warrendale, PA, USA). The sample was prepared by dropping the solution of nanoparticles onto carbon-coated copper grids for TEM observation. SEM samples were prepared by dropping the solution of nanoparticles onto carbon tape. The size analysis from TEM and SEM images was performed using ImageJ software (version 1.52a). Energy-dispersive X-ray spectroscopy (EDS) elemental mapping and line-profile elemental analysis were performed using a Noran system 7 by thermo scientific operated at 20 KV. UV-vis spectra were measured with a UV-2600 spectrophotometer (Shimadzu Corporation, Kyoto, Japan). The iron concentration was analyzed by using a Varian 820 Inductively Coupled Plasma Mass Spectrometer (ICP-MS) (Varian, Mulgrave VIC, Australia).

### 2.5. Stability Study

The in vitro Fe^3+^ leakage study was performed at different pHs. Typically, 2 mL of Amino-Fe-PDANPs at a concentration of 1 mg/mL were incubated in different pH media: (1) pH 6.5, (2) pH 6.8, (3) pH 7.5, (4) pH 8.0, respectively. At each time interval, 0.1 mL of medium was taken out and centrifuged to obtain supernatant. The leakage rate of Fe^3+^ was calculated by Fe^3+^ in the supernatant over the initial Fe^3+^ in the Amino-Fe-PDANPs.

The percentage of Fe^3+^ in nanoparticles was calculated by Fe mass obtained by ICP-MS in nanoparticles over the total mass of Amino-Fe-PDANPs. The solution of Amino-Fe-PDANPs was digested with aqua regia (VHCl:VHNO3 = 3:1) overnight. The solutions were diluted with 1% nitric acid solution before ICP-MS measurement.

To test the stability of Amino-Fe-PDANPs in a physiological environment, purified samples (0.2 mL) at a concentration of 1 mg/mL were dispersed in 2.0 mL phosphate buffer solution (PBS) (1mM) and 2.0 mL fetal bovine serum (FBS) solution, respectively. The photos of aqueous solution were taken over time.

### 2.6. MR Imaging Detection

The MRI performance of nanoparticles was evaluated by their longitudinal (*r*_1_) and transverse relaxivities (*r*_2_), which declare the ability of nanoparticles to alter *T*_1_ (spin-lattice relaxation) and *T*_2_ (spin-spin relaxation), respectively. The relaxation time was recorded at 25 °C using an NMI20-015V-I 0.5T MRI (NIUMAG, Shanghai, China) scanner. The analysis of the iron element was performed by ICP-MS. *T*_1_-weighted phantom images were acquired by a 3D *T*_1_-weighted gradient-echo pulse sequence. The nanoparticles were dispersed in aqueous solution with Fe^3+^ concentrations (by ICP-MS measurement) in the range of 0.0206 to 0.2081 mM. Milli-Q water was used as the control group. The *r*_1_ parameters were as follows: TR = 500 ms, TE = 20 ms, slice thickness = 3.0 mm, flip angle 90.0 degree and refoc flip angle 180.0 degree. The relaxation rate was plotted against the Fe^3+^ concentrations and the relaxivity was determined by a linear fit.

### 2.7. Photothermal Effect Measurement of Amino-Fe-PDANPs

The photothermal effect of amino-Fe-PDANPs was tested by recording the temperature changes with different concentrations (0, 2.26, 2.83, 3.77, 5.65, and 11.3 µg/mL) under irradiation by an 808 nm NIR laser (Power technology, Inc., Alexander, AR, USA) with the power density of 1.0 W cm^−2^ for 600 s. Pure water was used as a control group. The images and the data were obtained by using IR thermal camera (Infrared Cameras Inc., Beaumont, TX, USA). In order to evaluate the thermal stability of nanoparticles, the photothermal conversion experiment is conducted by two on-off cycles.

### 2.8. MTT Assay

3T3-L1 cells (ATCC) were plated at 50,000 cells per well in a 24 well cell culture plate (Corning Incorporated, Kennebunk, ME, USA) for 24 h until confluent. DMEM medium (Cytiva, Logan, UT, USA) was replaced, and cells were incubated with microparticles in DMEM for 24 h at 0, 10, 20, 50, 100, and 200 µL/mL concentrations with three replicates per concentration. Medium was removed and replaced with 250 µL of fresh medium and 50 µL of MTT stock solution (Life Technologies Corporation, Eugene, OR, USA) to incubate at 37 °C for 4 h. Three additional wells of medium and MTT stock solution were incubated without cells as a control. At the end of the incubation, all medium was removed, and 1 mL of DMSO (Sigma Aldrich, St. Louis, MO, USA) was added to each well and incubated at 37 °C for 10 min. The contents of the wells were collected, centrifuged to remove debris, and the DMSO supernatant was plated in a 96 well plate (Thermo Scientific, Rochester, NY, USA) with three replicates per sample and read in the ELx800 plate reader (BioTek, Winooski, VT, USA) for absorbance at 540 nm.

## 3. Results and Discussion

### 3.1. Synthesis and Characterization of Melanin-like Multifunctional Nanoparticles

**Figure 1:**
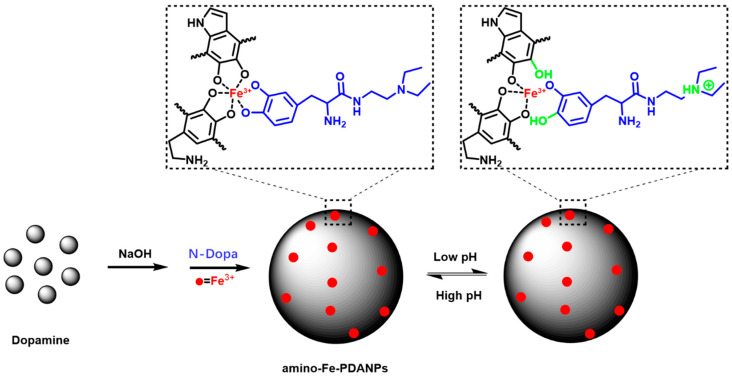
The synthetic process and pH-responsive properties of the amino-Fe-PDANP_S_.

The amino-Fe-PDANPs were synthesized by self-assembled polymerization of dopamine and N-Dopa in water. The synthetic route of the melanin-like nanoparticles (amino-Fe-PDANPs) and the pH-responsive property are illustrated in Figure 1. The possible chemical reactions are outlined in Figure S1. The size of the PDANPs can be tuned by the amount of NaOH solution. Typically, a size range from 70 nm to 350 nm can be obtained with a decrease of NaOH (Figure S2). The amino-Fe-PDANPs contain multifunctional components. The nanoparticles can serve as therapeutic agents which have a high photothermal effect. They are biocompatible due to their ability to degrade in the body. At the same time, N-Dopa is a pH-responsive compound in which the tertiary amine can be protonated under acidic conditions and promotes more protons into the internal structure. The Fe^3+^ cation is a *T*_1_ MRI metal chelated with catechol groups of polydopamine nanoparticles (PDANPs) and N-Dopa. The binding between Fe^3+^ and nanoparticles becomes weaker in a low pH environment due to the existence of more protons. Therefore, the amino-Fe-PDANPs could respond to pH and enhance magnetic resonance imaging.

**Figure 2:**
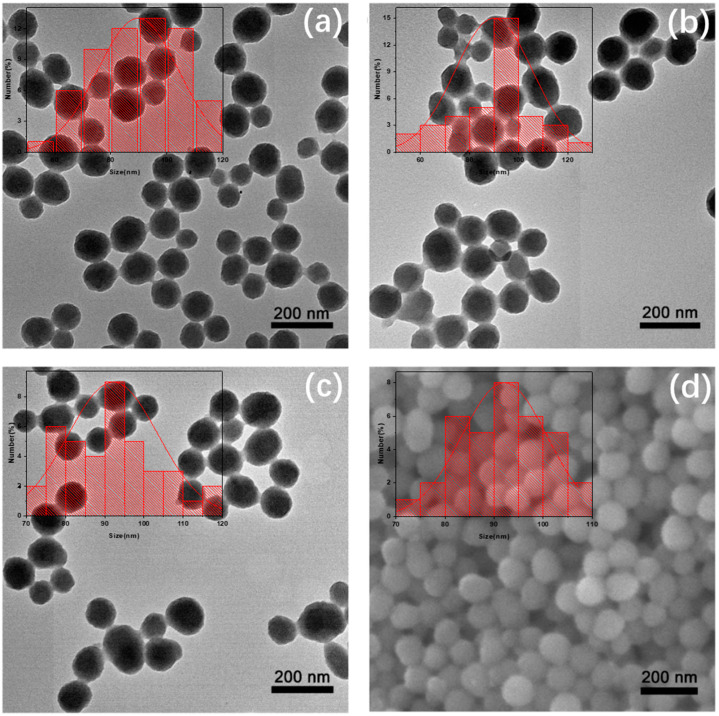
TEM images and nanoparticle size distribution histograms (insets) of (**a**) PDANPs, (**b**) amino-PDANPs, and (**c**) amino-Fe-PDANPs; (**d**) SEM image of amino-Fe-PDANPs.

The sizes and morphologies of the nanoparticles were investigated by transmission electron microscope (TEM). As shown in Figure 2, the size of PDANPs (Figure 2a), amino-PDANPs (Figure 2b), and amino-Fe-PDANPs (Figure 2c) were calculated to be 90 ± 15 nm, 90 ± 16 nm, and 92 ± 12 nm, respectively. The TEM measurement depicts that the size and morphology are almost the same during the synthesis. We can see that these nanoparticles are well dispersed in water solution. The scanning electron microscope (SEM) further confirms that the amino-Fe-PDANPS are spherical in shape, with an average diameter of 92 ± 9 nm (Figure 2d). The nano-sized spherical morphology and good water dispersity indicate great potential for further biomedical study.

**Figure 3:**
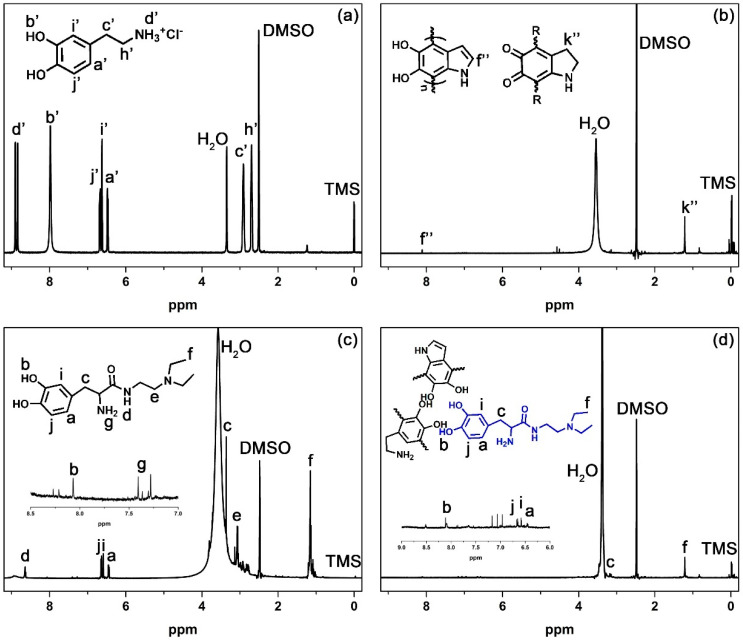
^1^H-NMR spectra of (**a**) dopamine, (**b**) PDANPs, (**c**) N-Dopa and (**d**) N-Dopa copolymerized polydopamine nanoparticles (amino-Fe-PDANPs).

To confirm the incorporation of N-Dopa, the ^1^H NMR spectra of the products were studied. As shown in Figure 3a, the peaks for dopamine monomer are 2.70 ppm (h’, C*H_2_*–NH_2_), 2.90 ppm (c’, Ar–C*H_2_*–CH_2_–NH_2_), 6.50 ppm (a’, Ar–*H*), 6.7 ppm (i’, Ar–*H*), 6.80 ppm (j’, Ar–*H*), 8.0 ppm (b’, Ar–O*H*), 8.9 ppm (d’, CH_2_–N*H_2_*) [36]. After polymerization, aromatic C–H signals in the region of 6–7.5 ppm are relatively weak in polydopamine nanoparticles (Figure 3b). The spectrum suggests that there are relatively few C–H signals of benzene ring in the structure. It is probably that hydrogens on the aromatic ring are substituted during the reaction to form polymers [37]. The result is consistent with the previous solid-state NMR study [33]. The structure of polydopamine nanoparticles varies and has many possibilities. Although it is not possible to assign all signals reliably, it can be assumed that the signal at 8.1 ppm corresponds to the indole group (f’’). As reported in the literature, the peak at 1.3 ppm (k”) may be related to aliphatic hydrogen in polydopamine [33]. In addition to the spectrum of N-Dopa (Figure 3c), the signals from polydopamine can be identified for amino-Fe-PDANPs (Figure 3d). The peak at 1.2 ppm is associate with CH_2_–C*H_3_* in N-dopa and can be identified in N-Dopa copolymerized polydopamine nanoparticles. Although there is a presence of a peak at 1.2 ppm for polydopamine, it was found that the intensity is much higher for amino-Fe-PDANPs. Besides, other peaks from N-Dopa are observed in N-Dopa copolymerized polydopamine, such as 6.58–6.68 (a, i, j), and 8.06 (b). The visibility of the aromatic hydrogen in amino-Fe-PDANPs could be attributed to the fact that N-Dopa has less polymerization activity.

**Figure 4:**
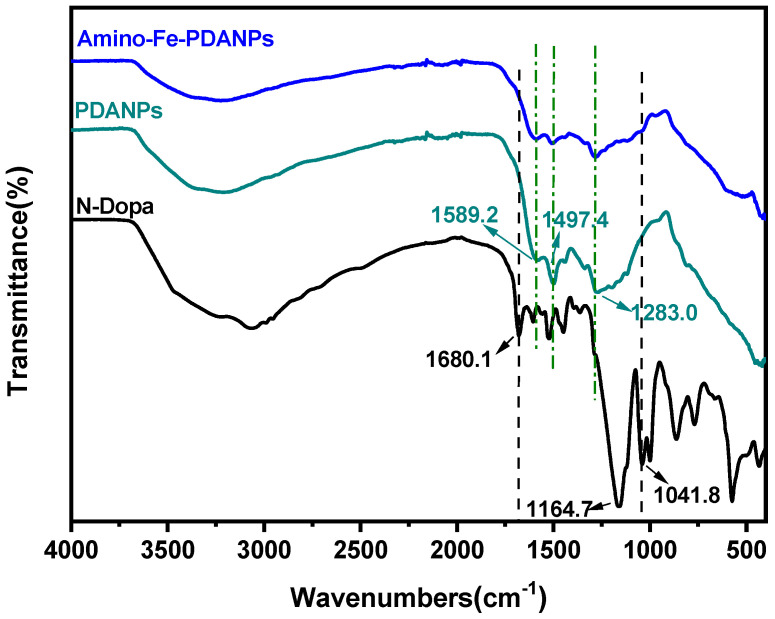
FTIR spectra of N-Dopa, PDANP_S_ and amino-Fe-PDANP_S_.

Fourier transform infrared (FTIR) spectra are showed in Figure 4. The FT-IR absorption for polydopamine nanoparticles exhibits a broad peak spanning from 3100 to 3500 cm^−1^ indicating the presence of hydroxyl groups. The peak from 1500 to 1700 cm^−1^ in the FT-IR spectrum corresponds to the indoline and indole structures, respectively. The peak of 1283.0 cm^−1^ is attributed to the stretching vibration of phenolic C–O [38]. For the N-Dopa doped nanoparticles, the FT-IR exhibits all three typical peaks as polydopamine (1283.0, 1497.4, 1589.2 cm^−1^). In addition, the peaks at 1164.7 and 1041.8 cm^−1^ are identified. The peaks are attributed to C–N peak in the structure of N-Dopa [39]. The peak at 1680.1 cm^−1^ is attributed to amide bond in N-Dopa. The result confirms that N-Dopa is incorporated into the structure.

The coordination of Fe^3+^ with nanoparticles was also confirmed by energy-dispersive X-ray spectroscopy (EDS) (Figure S3), which indicates that Fe^3+^ was incorporated into nanoparticles. The amount of Fe^3+^ bound to the nanoparticles was measured using ICP-MS as 1.6 mg of Fe per 100 mg of nanoparticles. The chelation of Fe^3+^ with nanoparticles is strong. There was a negligible amount of Fe^3+^ (less than 0.1% comparing to the total Fe^3+^) that could leak out after the nanoparticle solutions were incubated for 48 h at various pHs (Figure S4). The colloidal stability of amino-Fe-PDANPs was further evaluated in PBS and FBS (Figure S5). No precipitate was observed after incubation for 24 h.

### 3.2. pH-Responsive Relaxivity Properties of Amino-Fe-PDANPs

**Figure 5:**
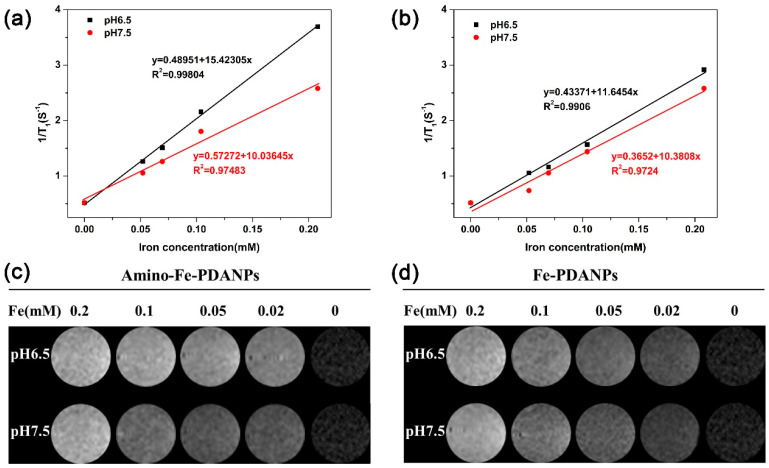
The plots of 1/T_1_ as a function of the molar concentration of Fe^3+^ in the solution under pH 6.5 and pH 7.5 for (**a**) the amino-Fe-PDANPs and (**b**) Fe-PDANPs; *T*_1_-weighted MRI phantom results from (**c**) amino-Fe-PDANP_S_ and (**d**) Fe-PDANP_S_ as a function of the molar concentration of Fe^3+^ in the solution under different pH values. Water is the control group.

To examine the feasibility of amino-Fe-PDANPs as pH-responsive *T*_1_-weighted MRI contrast agents, the longitudinal (*r*_1_) relaxivity was measured under various pH levels. As shown in Figure 5, the relaxivity values (the slopes of each plot, mM^−1^ s^−1^) were determined based on the relaxation rates (reciprocal values of relaxation times) against the amount of bound Fe^3+^ ions (1/T vs. [Fe^3+^]). The *r*_1_ relaxivity of the amino-Fe-PDANPs is 10.0 mM^−1^ s^−1^ at pH 7.5. When the pH value decreases to pH 6.5, the value of *r*_1_ increases to 15.4 mM^−1^ s^−1^ (Figure 5a). This trend is higher than those of reported contrast agents on pH dependency [40]. In contrast, we also synthesis the reagent of Fe-PDANPs without N-Dopa and explore the pH-responsive relaxivity. With pH values adjusted from 7.5 to 6.5, we notice that the value of *r*_1_ increases from 10.4 mM^−1^ s^−1^ to 11.6 mM^−1^ s^−1^, respectively (Figure 5b). The *r*_1_ value of amino-Fe-PDANPs has been increased more than that of Fe-PDANPs with the pH values decreased from 7.5 to 6.5.

The *T*_1_-weighted MR images with the changes of pH are studied at different iron concentrations (Figure 5c). It is apparent to see from these images that the nanoparticles present a concentration dependent contrast enhancement. Nevertheless, by comparing the contrast effect of nanoparticles under pH 7.5, we can observe a bright signal enhancement even at a low concentration in pH 6.5. For the Fe-PDANPs group, there is a slight signal change with the decrease of pH values (Figure 5d). As expected, the *T*_1_ phantom imaging results are consistent with the *r*_1_ measurement. Therefore, the amino-Fe-PDANPs can act as highly efficient *T*_1_ contrast agents with pH-responsive properties. The relaxivity study indicates the great potential of amino-Fe-PDANPs for stimuli-responsive applications such as the tumor microenvironment.

### 3.3. Photothermal Therapy Effect

**Figure 6:**
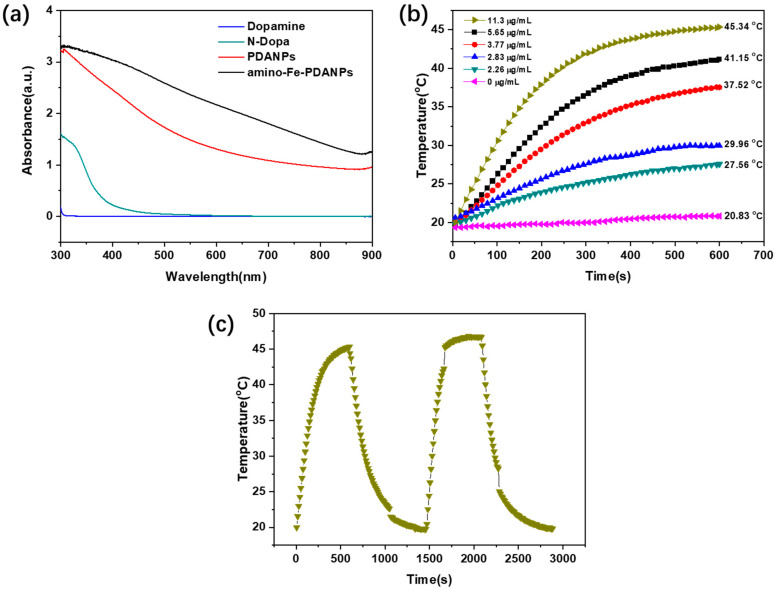
(**a**) UV–vis absorption spectra of Dopamine, N-Dopa, PDANP_S_ and amino-Fe-PDANP_S_, the concentrations are 1 mg mL^−1^; (**b**) Temperature elevation of water and amino-Fe-PDANP_S_ aqueous solutions with different concentrations over the irradiation time; (**c**) The repeated photothermal response of the amino-Fe-PDANP_S_ aqueous solution.

The UV-Vis spectra of dopamine, PDANPs, N-Dopa, and Amino-Fe-PDANPs are shown in Figure 6a. The dopamine monomer and N-Dopa have no absorption in the NIR region, which means that they cannot be used for photothermal reagents. In contrast, both PDANPs and amino-Fe-PDANPs aqueous solutions exhibit broad absorption in the range of 400−900 nm. It is attributed to the oxidation of dopamine into dopachrome and dopaindole. The following self-polymerization process can lead to a pronounced absorption extending from visible to NIR wavelengths [33]. Comparing to the PDANPs, amino-Fe-PDANPs present a strong NIR absorption after being incorporated with N-Dopa. Then, the PTT performance of amino-Fe-PDANPs is studied. The amino-Fe-PDANPs were dispersed in water at concentrations ranging from 2.26 to 11.3 μg/mL, pure water was used as a control. As expected, we can observe an obvious temperature increase through the heating curves as irradiated with an 808 nm laser. (Figure 6b). The photothermal effect is concentration dependent. Within 600 s, the temperature of amino-Fe-PDANPs with a concentration of 11.3 μg/mL raises from 19.97 to 45.34 °C, which is high enough to cause cell death [10], while almost no significant temperature change is observed for Milli-Q water. Meanwhile, the amino-Fe-PDANPs are stable during photothermal therapy. After the laser is turned off after 600 s and the temperature decreases from 45.34 to 19.85 °C, the solution temperature can be heated to 45 °C again by radiation of 808 nm laser (Figure 6c). All these results suggest that amino-Fe-PDANPs are potential photothermal reagents to convert NIR laser energy to hyperthermia. The amino-Fe-PDANPs are stable and can be used for repeated photothermal therapy.

### 3.4. Cytotoxicity Studies

**Figure 7:**
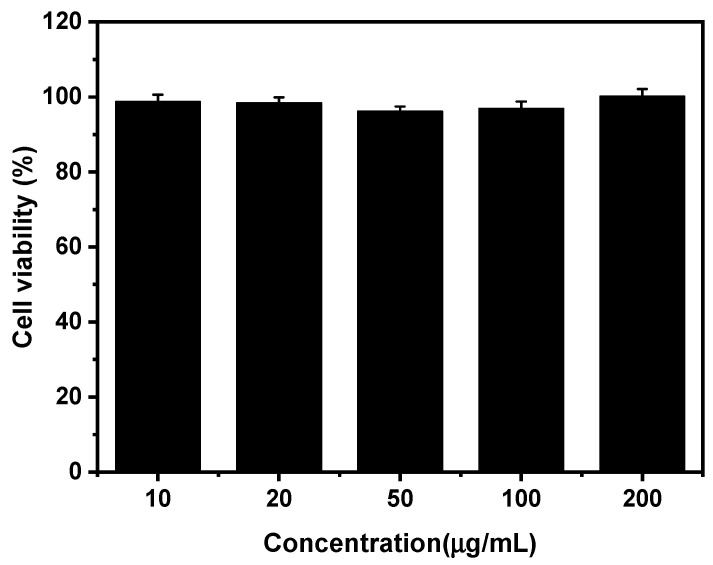
The cell viability of 3T3-L1 cells incubated with different concentrations of amino-Fe-PDANPs for 24 h.

The cytotoxicity of amino-Fe-PDANPs was further evaluated by using MTT assay. The cells treated with a culture medium were used as a control. The result is shown in Figure 7. It is demonstrated that there is no obvious cell viability change after 24 h incubation when the concentration is as high as 200 µg mL^−1^. The cytotoxicity study indicates the excellent biocompatibility of amino-Fe-PDANPs for further application in the biomedical field.

## 4. Conclusions

In summary, we successfully designed and synthesized a novel contrast agent, amino-Fe-PDANPs, for *T*_1_-weighted MR imaging enhancement and PTT treatment. The obtained nanoparticles had a size of about 90 nm, and the structure was characterized by ^1^H-NMR, FTIR, TEM, SEM, and EDS. In addition, the incorporation of N-Dopa enabled the nanoparticles to respond to the change of pH. The tertiary amine group on N-dopa can be protonated under the acid environment. The *r*_1_ relaxivity of amino-Fe-PDANPs increased from 10.0 to 15.4 mM^−1^ s^−1^ when pH changed from 7.5 to 6.5. By comparison, the *r*_1_ increased from 10.4 to 11.6 mM^−1^ s^−1^ for Fe-PDANPs. Furthermore, amino-Fe-PDANPs exhibited a high photothermal effect. Taken together, the highly biocompatible amino-Fe-PDANPs could be used as a theranostic agent for MRI-guided photothermal therapy and expecting a great promise for further study.

## Data Availability

The data are available within the manuscript and the corresponding supporting information file.

## Supplementary Materials

The following are available online at https://www.mdpi.com/article/10.3390/nano11082107/s1, Figure S1: The possible chemical reactions in the synthesis of PDANPS and amino-Fe-PDANPS, Figure S2: The TEM images of PDANPs synthesized using different amounts of NaOH, Figure S3: EDS of amino-Fe-PDANPS. Figure S4: The iron concentration leaked out of the solution, Figure S5: Photographs of amino-Fe-PDANPs after incubation with FBS and PBS.
