# Supplementary material for: Synthesis of Biomimetic Melanin-Like Multifunctional Nanoparticles for pH Responsive Magnetic Resonance Imaging and Photothermal Therapy
Source: Nanomaterials (Basel). 2021 Aug 19;11(8):2107. doi: 10.3390/nano11082107 (PMC8400264; doi:10.3390/nano11082107)
Supplement: Supplementary file 1 [file nanomaterials-11-02107-s001.zip › nanomaterials-1306395-supplementary.pdf]

nanomaterials

Electronic Supporting Information

Synthesis of Biomimetic Melanin-like Multifunctional Nanoparticles for pH Responsive Magnetic Resonance Imaging and Photothermal Therapy

Jing Qu [1], Devin Guillory [1], Pohlee Cheah [1], Bin Tian [1], Jie Zheng [2], Yongjian Liu [2], Courtney Cates [3], Amol V. Janorkar [3] and Yongfeng Zhao [1,*]

[1] Department of Chemistry, Physics and Atmospheric Science, Jackson State University, Jackson, MS 39217, USA; jing.qu@students.jsums.edu (J.Q.); dmcguillory@yahoo.com (D.G.); pohlee.cheah@jsums.edu (P.C.); bin.tian@jsums.edu (B.T.); yongfeng.zhao@jsums.edu (Y.Z.)
[2] Mallinckrodt Institute of Radiology, Washington University School of Medicine, St. Louis, MO 63110, USA; zhengj@wustl.edu (J.Z.); yongjianliu@wustl.edu (Y.L.)
[3] Department of Biomedical Materials Science, School of Dentistry, University of Mississippi Medical Center, Jackson, MS 39216, USA; ccates@umc.edu (C.C.); ajanorkar@umc.edu (A.J.)
* Correspondence: yongfeng.zhao@jsums.edu

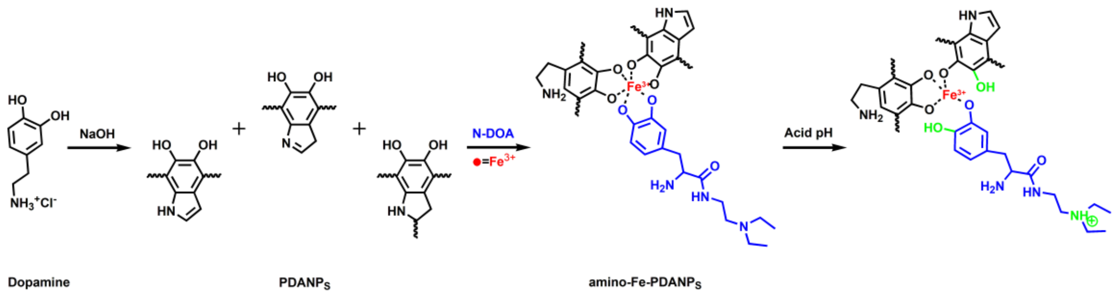

Figure S1. The possible chemical reactions in the synthesis of PDANPs and amino-Fe-PDANPs.

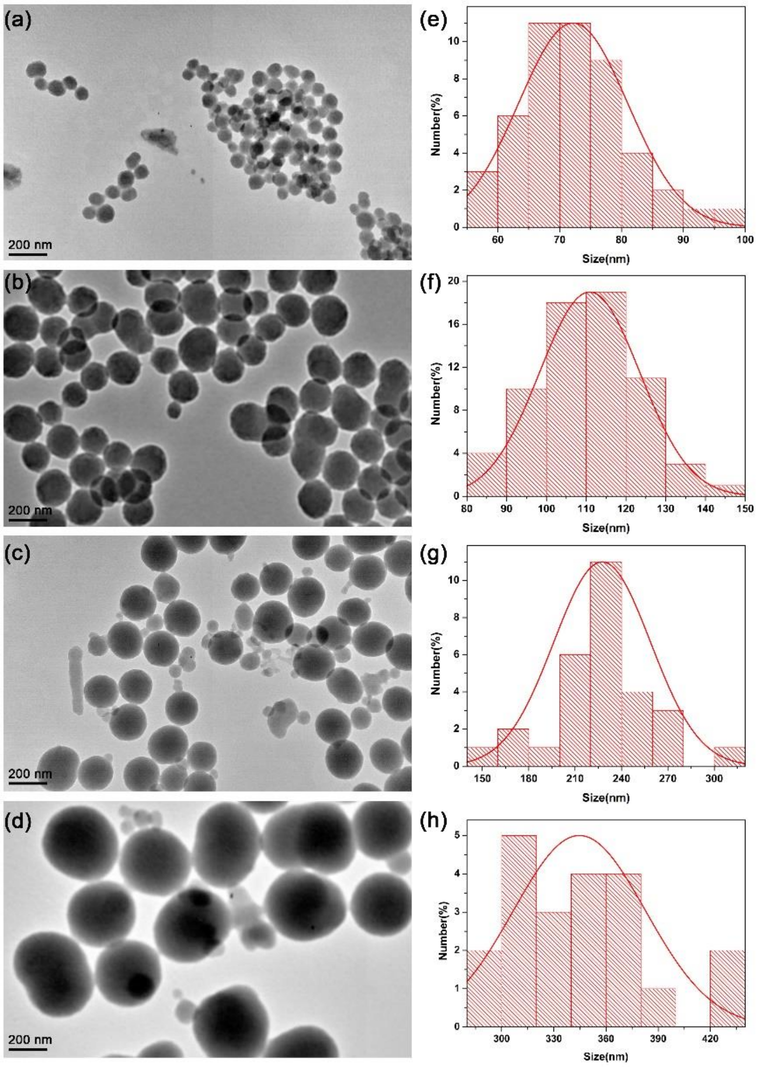

Figure S2. The TEM images of PDANPs synthesized using different amount of NaOH. The average size for each NaOH amount are (a) NaOH 45.5 μL: 72.2 ± 8.8 nm, (b) NaOH 38 μL: 110.9 ± 12.6 nm, (c) NaOH 32 μL: 227.5 ± 31.3 nm, and (d) NaOH 28 μL: 344.7 ± 38.0 nm. (e-h) are nanoparticle size distribution histogram corresponding to figure a-d, respectively.

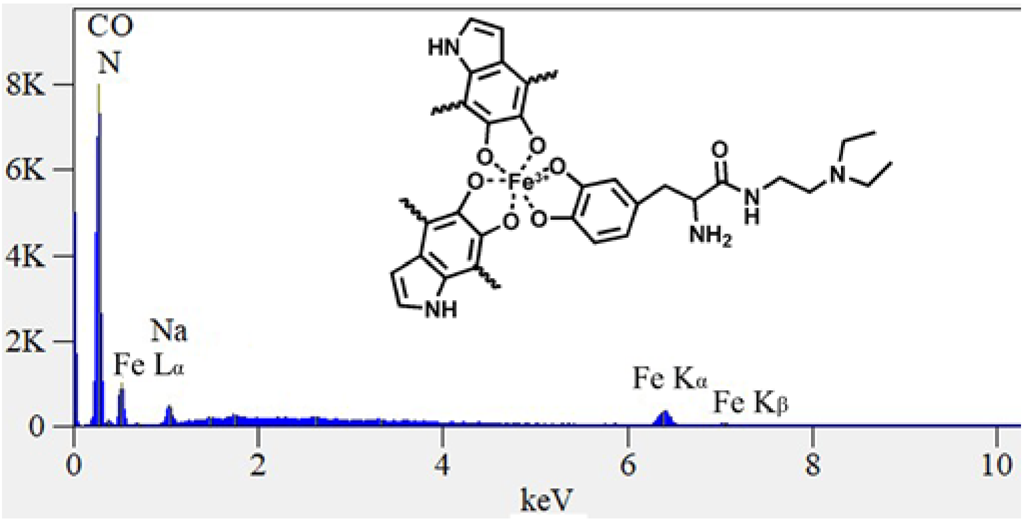

Figure S3. EDS of amino-Fe-PDANPs.

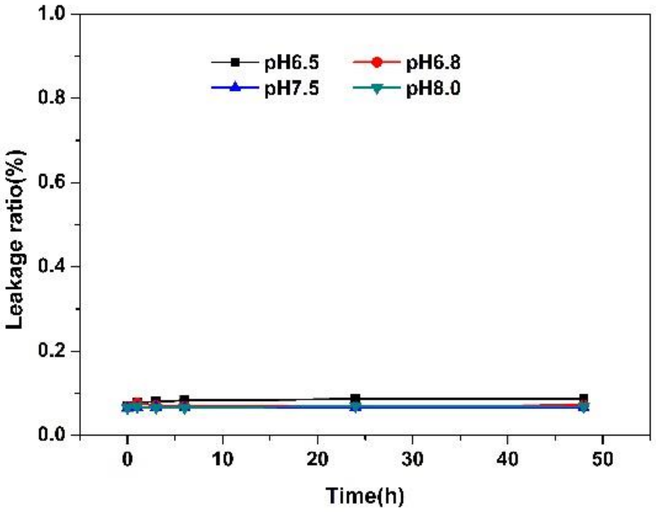

Figure S4. The iron percentage leaking out of the nanoparticles at various pHs.

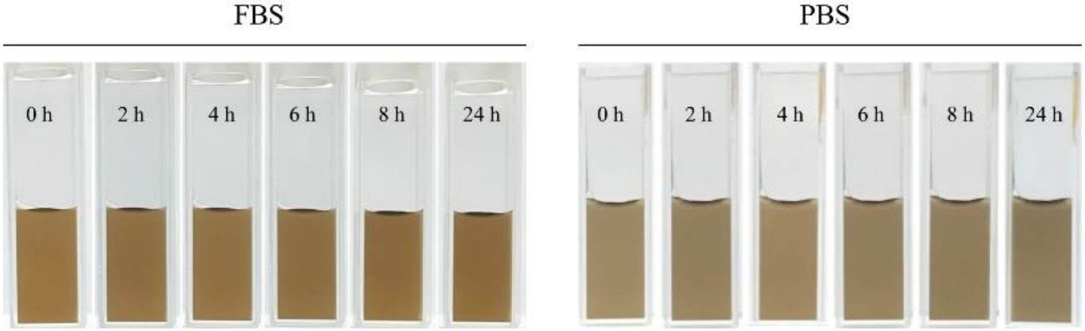

Figure S5. Photographs of amino-Fe-PDANPs after incubation with FBS and PBS for 24 h.
